# A new KAT on the block rewrites the epigenetic script in menin inhibitor‐resistant leukemia

**DOI:** 10.1002/hem3.70265

**Published:** 2025-11-19

**Authors:** Yizhou Huang, Charles E. de Bock

**Affiliations:** ^1^ Children's Cancer Institute Lowy Cancer Research Centre Randwick New South Wales Australia; ^2^ School of Clinical Medicine, Faculty of Medicine UNSW Sydney Sydney New South Wales Australia

Chromosomal translocations involving the mixed lineage leukemia (also known as *MLL* or *KMT2A*) gene occur in approximately 40% of children with acute myeloid leukemia (AML) under the age of 3. The fusion partners of *MLL* can vary widely with over 130 different ones identified to date. These fusions result in constitutive activation of MLL and ultimately drive the overexpression of key downstream target genes including *MEIS1* and *HOXA* cluster genes that leads to leukemia development. Critically, *MLL*‐rearranged (*MLL*‐r) leukemias all depend on menin for growth and survival that in turn has driven the development of menin inhibitors (e.g., revumenib) currently being evaluated in numerous clinical trials. I refer the reader to two excellent recent reviews on the biology of *MLL*‐r AML and early clinical trial data.^1^, ^2^

Another common chromosomal translocation in AML is the one involving the *NUP98* gene, which has up to 30 distinct fusion partners. These have been shown to drive transcription condensates in the nucleus as well as changes in chromatin architecture that are thought to play an important role in rewiring cells toward leukemia development.^3^ Importantly, NUP98 fusion proteins interact with wild‐type MLL (KMT2A) complexes, and this also drives high expression of *MEIS1* and *HOXA* cluster genes. Notably, *NUP98*‐rearranged (*NUP98*‐r) AML cases are also very sensitive to menin inhibitors.^4^

Nevertheless, just like other targeted therapies, resistance mechanisms have now been identified in AML cases treated with menin inhibitors. These include mutations in the drug pocket of menin around amino acid residues M327, G331, T349, and S160 that prevent the binding of menin inhibitors. Alternative mechanisms include epigenetic wiring such as loss of components of the PRC1.1 complex.^2^ Interestingly, a new study showed that chemotherapy could also render *MLL*‐r leukemia more resistant to menin inhibition without prior exposure to menin inhibitors.^5^ Therefore, combination strategies are needed to increase the chances of complete remission and cure.

Two new studies^6^, ^7^ have now been published back‐to‐back in *Cancer Discovery* addressing this very issue, with both papers looking at the efficacy of combining menin inhibitors with the newly developed orally bioavailable KAT6A/B selective inhibitor PF‐9363. Both KAT6A and KAT6B are members of the MYST (MOZ/KAT6A, Ybf2/Sas3, Sas2, and Tip60) family of histone acetyltransferases (HATs) that also includes KAT5, KAT7, and KAT8. These all differentially regulate the acetylation of lysine residues on histone 3, with KAT6A and KAT6B driving H3K23Ac whilst KAT7 is responsible for H3K14Ac for example. Whilst this family is important in regulating chromatin structure and normal developmental processes, *KAT6A* is an oncogene in many human cancers, including leukemia, breast cancer, and hepatocellular cancer and therefore a candidate for cancer therapy.^8^ One of the new promising inhibitors and first in class orally available KAT6A/B selective inhibitor is PF‐9363,^9^ albeit this can also inhibit other MYST family members at higher concentrations with a dose dependent hierarchy of KAT6A/B > KAT7 >> KAT8 > KAT5.^10^

In the new study by Michmerhuizen et al.,^7^ the authors investigated *NUP98*‐r AML and identified novel interacting partners of NUP98 fusion proteins. Using a proteomics technique called Rapid Immunoprecipitation Mass spectrometry of Endogenous proteins (RIME), they discovered that members of the MYST HAT family including KAT6A and KAT7, the scaffold protein MEAF6, and the chromatin reader BRPF1 interacted with NUP98 fusion proteins. Further evidence for the interaction between MYST family members and NUP98 fusion proteins came from the analysis of transcription condensates within the nucleus. These were found to be associated with the histone H3 post‐translational modification H3K23Ac, which is catalyzed by KAT6A/B.

To complement these findings, the researchers then performed an in vivo CRISPR/Cas9 screen to functionally test whether 337 epigenetic regulators influenced cell fitness in a *NUP98::KDM5A*‐driven leukemia model. Among the most depleted guide RNAs were those targeting BRPF1, an epigenetic reader and member of the MYST family HAT complex. Together, these data support a model in which NUP98 fusion proteins form a complex not only with MLL (KMT2A) and menin but also with KAT6A‐ and KAT7‐containing HAT complexes. These complexes deposit histone H3 acetylation marks, sustaining the expression of key downstream oncogenes such as *MEIS1*, thereby promoting leukemic cell proliferation (Figure 1).

**Figure 1:**
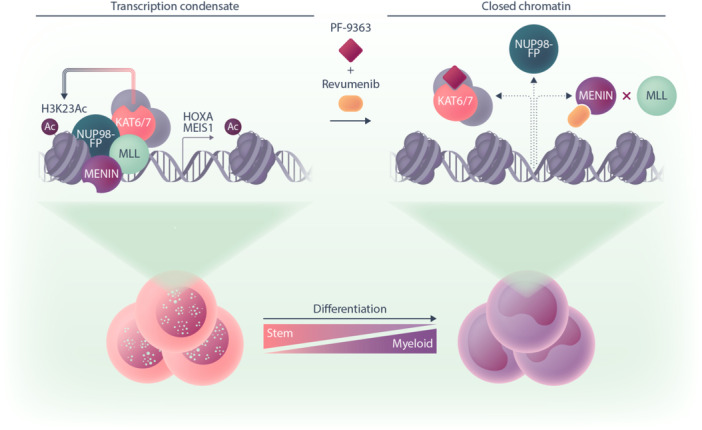
**Combination treatment of *NUP98*‐rearranged (NUP98‐r) acute myeloid leukemia (AML) with the menin inhibitor revumenib and KAT6/7 inhibitor PF‐9363 drives differentiation.** NUP98‐fusion proteins (NUP98‐FP) form transcription condensates in the nucleus where fusion proteins interact with mixed lineage leukemia (MLL), menin, and members of the MYST family histone acetyltransferase (HAT) complex including KAT6 that deposits H3K23Ac or KAT7. This drives expression of key downstream oncogenes such as *MEIS1* and *HOXA* cluster genes. Treatment of cells with revumenib dissociates the MLL—menin interaction and low concentrations of PF‐9363 block the catalytic activity of KAT6 that together results in removal of the NUP98‐FP complex from DNA, downregulation of *MEIS1* and *HOXA* cluster genes, chromatin condensation, and cellular differentiation.

This posed the inevitable question, whether these newly identified interactions and dependencies for NUP98 fusion proteins could be exploited therapeutically? Moreover, with menin inhibitors now in clinical trial for *NUP98*‐r AML, would combining menin inhibitor with KAT6/7 inhibitor be synergistic?

Using a range of in vitro experimental models, treatment of cells harboring *NUP98::HOXA9*, *NUP98::KDM5A*, or *NUP98::NSD1* fusions with PF‐9363 alone reduced cell viability and increased CD11b expression, suggesting myeloid differentiation (Figure 1). Importantly, CD34⁺ hematopoietic stem cells did not exhibit a comparable loss of viability following PF‐9363 treatment, indicating a degree of selectivity for leukemic cells.

Moving to in vivo patient‐derived xenograft (PDX) models carrying either *NUP98::HOXA13* or the more common *NUP98::NSD1* fusions, PF‐9363 treatment reduced leukemia burden and prolonged survival. When combined with the menin inhibitor revumenib (SNDX‐5613) in a *NUP98::KDM5A*‐driven acute megakaryocytic leukemia PDX model, the combination increased CD41 expression, consistent with enhanced differentiation, although bone marrow clearance was not improved compared to single‐agent treatment.

More promising efficacy was observed in a *NUP98::NSD1* PDX model resistant to menin inhibition, where the PF‐9363 and revumenib combination induced tumor regression, rather than merely delaying progression, relative to either agent alone. These findings support the potential clinical benefit of combining KAT6/7 and menin inhibitors in AML cases exhibiting resistance to menin‐targeted therapy.

The ability of PF‐9363 to overcome menin inhibitor resistance is further demonstrated in the complementary study by Gordon et al.,^6^ which focused on *MLL* fusion–driven and *NPM1c*‐mutant AML. The authors first showed that single‐agent concentrations of PF‐9363 must be sufficiently high to inhibit both KAT7 and KAT6A/B to impair leukemic cell growth. In *MLL::AF6* OCI‐AML2 cell line resistant to the menin inhibitor VTP50469, only genetic loss of *KAT7* and not *KAT6A/B* significantly reduced cell fitness. Moreover, an *MLL::AF4* PDX model harboring menin mutations G331D and T349M that drove resistance remained sensitive to single‐agent PF‐9363 in vivo (Figure 2).

**Figure 2:**
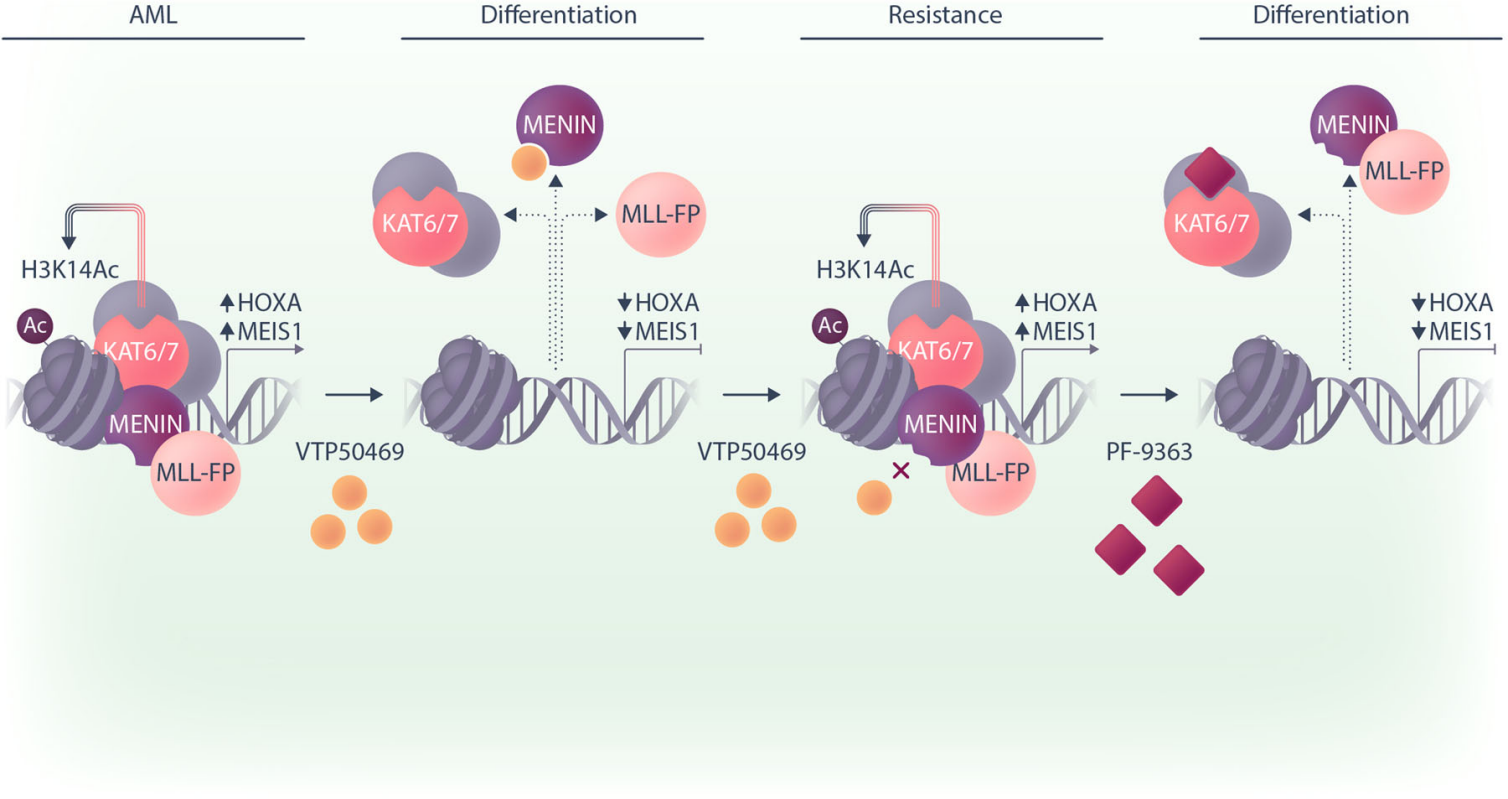
***Mixed lineage leukemia* (*MLL*)‐rearranged (*MLL*‐r) acute myeloid leukemia (AML) that is resistant to menin inhibitors remains sensitive to PF‐9363.** MLL‐fusion proteins (MLL‐FP) drive leukemia growth and survival of AML cells by upregulating *MEIS1* and *HOXA* genes. The menin‐MLL‐FP complex is also associated with KAT6/7 albeit mostly KAT7 that deposits H3K14Ac marks. When cells are treated with menin inhibitors (e.g., VTP50469), the MLL‐FP complex dissociates, resulting in cellular differentiation. However, chronic exposure to menin inhibitors over time drives resistance including binding pocket changes that prevent VTP50469 binding. Using higher concentrations of PF‐9363 to inhibit KAT7, a dependency in *MLL*‐r AML, can overcome menin inhibitor resistance, dissociate the complex, and drive cellular differentiation.

In contrast to the findings of Michmerhuizen et al.,^7^ combination treatment of an *MLL::AF6* AML PDX with SNDX‐5613 (revumenib) and PF‐9363 resulted in marked clearance of leukemic blasts from the bone marrow and enhanced differentiation. Notably, this differentiation was so profound that surviving cells from combination‐treated mice lost all leukemia‐initiating potential when serially transplanted into recipient mice. The authors, however, cautioned that if such potent differentiation effects were recapitulated in patients, they could trigger AML differentiation syndrome and a cytokine release “storm”, underscoring the need for careful clinical monitoring during future trials.

Together, these two studies exemplify how fundamental discovery‐based research ranging from immunoprecipitation experiments that uncover novel protein–protein interactions to elegant CRISPR/Cas9 genetic screens can reveal mechanistic dependencies that inform rational combination therapies. Specifically, they provide a strong rationale for combining complementary epigenetic therapies to target high‐risk AML. The question now is whether additional resistance mechanisms will result from this combinatorial approach. Nevertheless, from a clinical standpoint over the short term, both revumenib and the new PF‐9363 are orally bioavailable agents and if approved for AML, will offer patients a more convenient and less invasive treatment option compared with intravenous regimens that require repeated hospital visits.

## AUTHOR CONTRIBUTIONS

**Yizhou Huang**: Writing—original draft; writing—review and editing; conceptualization. **Charles E. de Bock**: Writing—original draft; writing—review and editing; conceptualization.

## CONFLICT OF INTEREST STATEMENT

The authors declare no conflicts of interest.

## FUNDING

No funding was received for this publication.

## Data Availability

Data sharing is not applicable to this article as no datasets were generated or analyzed during the current study.
